# Strengthening Preparedness for Arbovirus Infections in Mediterranean and Black Sea Countries: A Conceptual Framework to Assess Integrated Surveillance in the Context of the One Health Strategy

**DOI:** 10.3390/ijerph15030489

**Published:** 2018-03-10

**Authors:** Maria Grazia Dente, Flavia Riccardo, Gloria Nacca, Alessia Ranghiasci, Camille Escadafal, Lobna Gaayeb, Miguel Angel Jiménez-Clavero, Jean-Claude Manuguerra, Marie Picard, Jovita Fernández-Pinero, Elisa Pérez-Ramírez, Vincent Robert, Kathleen Victoir, Silvia Declich

**Affiliations:** 1 Istituto Superiore di Sanità, 00161 Rome, Italy; flavia.riccardo@iss.it (F.R.); gloria.nacca@iss.it (G.N.); alessia.ranghiasci@iss.it (A.R.); silvia.declich@iss.it (S.D.); 2 Institut Pasteur, 75015 Paris, France; camille.escadafal@finddx.org (C.E.); lobna.gaayeb@pasteur.fr (L.G.); jean-claude.manuguerra@pasteur.fr (J.-C.M.); kathleen.victoir@pasteur.fr (K.V.); 3 FIND (Foundation for Innovative New Diagnostics), 1202 Geneva, Switzerland; 4 Centro de Investigación en Sanidad Animal-Instituto Nacional de Investigación y Tecnología Agraria y Alimentaria (INIA-CISA), 28040 Madrid, Spain; majimenez@inia.es (M.A.J.-C.); fpinero@inia.es (J.F.-P.); elisaperezramirez@gmail.com (E.P.-R.); 5 CIBER Epidemiología y Salud Pública (CIBERESP), 28029 Madrid, Spain; 6 Institut de Recherche pour le Développement (IRD), UMR Mivegec IRD-CNRS-Univ. Montpellier, 34394 Montpellier CEDEX 5, France; marie.picard@ird.fr (M.P.); vincent.robert@ird.fr (V.R.)

**Keywords:** One Health, integrated surveillance, West Nile virus (WNV), chikungunya virus (CHKV), dengue virus (DENV), Rift Valley fever virus (RVFV)

## Abstract

In the context of One Health, there is presently an effort to integrate surveillance of human, animal, entomological, and environmental sectors. This aims to strengthen the prevention of, and preparedness against, arbovirus infections, also in the light of environmental and climate changes that could increase the risk of transmission. However, criteria to define integrated surveillance, and to compare different systems, still need to be identified and tested. We conducted a scoping review to identify and examine surveillance systems for West Nile virus (WNV), chikungunya virus (CHKV), dengue virus (DENV), and Rift Valley fever virus (RVFV), which involve human, animal, entomological, and environmental sectors. We analyzed findings using a conceptual framework we developed for this purpose. The review highlights that the criteria proposed in the conceptual framework to describe integrated surveillance are consistently reported in the context of studies and programs related to integrated surveillance of the selected arboviral diseases. These criteria can facilitate the identification and description of operationalized One Health surveillance.

## 1. Introduction

Since 2000, a number of meetings have taken place and documents produced that have highlighted the need for a more proactive and comprehensive approach to problems that affect the health of humans, animals, and the general environment. Such an approach is not new, but it is advocated for with growing urgency due to the increasing incidence of diseases that have the potential of creating a large economic impact, impairing human health and provoking losses of environmental diversity [1,2,3,4,5].

Given the alarming increase of human pathogen emergence from animal reservoirs, the rationale behind the concept of One Health (OH), namely the promotion of a more harmonized and integrated approach to monitor, investigate, plan for, and react to mitigate zoonotic disease risks, is promising and is receiving international consensus [6,7,8,9,10,11].

The concept of OH, as developed especially in the last decade [12,13,14,15], is defined as the collaborative effort of multiple disciplines to attain optimal health for people, animals, and our environment [16,17].

Many infectious disease pathogens are multi-host, with cross-species and multi-sectoral impacts. It is therefore not surprising that OH was conceived originally in the context of veterinary science and infectious disease. More recently, antimicrobial resistance is also being attributed a strong OH label given the cross-species drivers for this serious challenge to modern medicine and emergency preparedness [18,19].

However, despite all efforts of cooperation between human and animal health, silo thinking persists, particularly in the public health sector that hesitates to perceive the advantages of using an OH approach [1,11,18,20].

One Health surveillance is the latest conceptual tool being proposed to prove the added value of the OH concept and to ultimately reduce the risks of infectious diseases at the animal–human–ecosystem interfaces. One Health surveillance consists of the systematic collection, validation, analysis, and interpretation of data and the dissemination of information collected on humans, animals, and the environment to inform decisions for more effective, evidence- and system-based health interventions. One of the four types of inter-sectoral collaboration that can support improvements in the technical and/or economic efficiency of OH risk mitigation programs is considered [6,10,21].

At this stage, sporadic national success stories exist in implementing OH surveillance that can serve as examples for further implementation [22,23], and integrated surveillance systems have worked in specific situations and contexts. International initiatives have been launched and supported by the Food and Agriculture Organization (FAO), the World Health Organization (WHO), and the World Organization for Animal Health (OIE) [24]. Additionally, some methodologies for the aggregation of existing databases at the human–animal interface have been tested (for example, the Joint FAO–OIE–WHO Global Early Warning System for health threats and emerging risks at the human–animal–ecosystems interface (GLEWS) database) [25].

Although there are increasing demands on disease surveillance, preparedness, response, and control in the OH context, no standardized methodology exists for controlled comparative cost-efficacy studies and quantitative evaluation of OH activities. New interesting initiatives are ongoing [26], but criteria and methods to describe and assess existing levels of integration of surveillance for specific pathogens (including a wide range of individual and environmental exposures) to facilitate the evaluation of the impact and the added value of OH, are still to be defined and tested [27].

Moreover, the effectiveness of such integrated surveillance should be compared with effectiveness in more segregated systems. To facilitate this, the development of a comprehensive conceptual framework is also suggested and recommended [6,28,29].

The OH strategy is receiving attention in geographical areas where priority is given to the surveillance and control of vector-borne diseases (VBDs). The surveillance and control of VBDs, with their complex life cycles often involving human and animal hosts and transmitted by vectors that are strongly influenced by the environment, benefit intuitively from the OH strategy, although important efforts are still needed to operationalize and move “the strategy” forward in areas where multiple and inter-sectoral inputs are essential [30].

In line with this, in 2009, the EpiSouth Network, implementing infectious disease surveillance and preparedness activities in the Mediterranean Basin and in the Balkans [31], created a directory of Human Public Health (HPH) and Veterinary Public Health (VPH) Officials for zoonoses [32] in order to facilitate the surveillance of zoonosis in the framework of OH. The network also identified the need to establish national networks for preparedness and response, in line with the International Health Regulations, including the HPH and VPH authorities and all the recognized actors of the process [33].

To continue and reinforce the EpiSouth Network’s strategy, the European project MediLabSecure was launched (2014–2018) with the aim to improving surveillance and monitoring of emerging arboviral diseases in the Mediterranean basin and Black Sea regions [34]. This network comprises 55 laboratories and 19 public health institutions/ministries of health in 19 non-EU countries in the Mediterranean and Black Sea regions.

This OH project is developed through the transdisciplinary interaction of four sectors (sub-networks)—human virology, animal virology, medical entomology, and public health—to enhance preparedness and response to emerging arboviral diseases and to improve integration of surveillance across sectors.

This article describes the review that we performed, together with other studies, to facilitate the description and comparison of integrated surveillance systems of arboviral diseases.

## 2. Materials and Methods

We performed a review of scientific and gray literature as proposed by Arksey et al. [35], taking also into account the methodology proposed by Khan et al. [36], and search strategies as presented by Relevo et al. [37] and by DeLuca et al. [38] (Table S4).

The general objective of this review was to gather documented experiences reporting criteria that enable one to consistently describe the integrated surveillance of arboviral diseases. Given the specificity of integrated surveillance activities for the different pathogens, this review was defined on the basis of the working priorities for the MediLabSecure network: mosquito-transmitted arboviruses, which have caused locally transmitted (autochthonous) cases of disease (endemic/sporadic) in the EU, Mediterranean, and Black Sea regions. For this reason, we focused on WNV, CHKV, DENV, and RVFV.

To meet this general objective, we identified the following two specific objectives: (i) to analyze publications describing existing surveillance systems of arboviral diseases, integrating human and/or animal and/or medical entomology and/or environmental surveillance, and (ii) to identify levels and criteria that can define a surveillance system as “integrated”.

In order to do this, we developed a framework (Table 1) to assess existing levels of integration between human/animal/entomological/environmental surveillance for a specific pathogen on the basis of existing operational protocols and procedures [39].

We validated this proposed framework in 2015 with a survey asking the national experts of the different sectors of the MediLabSecure network to describe at what levels the surveillance systems for arboviral diseases, that they considered integrated in their country, were inter-operating [40].

This framework allows for an assessment of the levels of integration through specific criteria, which are presumed to be connected with the operationalization of the OH approach at the country level. For example, the integration at policy and institutional level is expected to facilitate harmonization of activities and programs, leading to a more efficient use of resources; integration at data collection and analysis level is expected to facilitate inter-sectoral coordination and planning and indicates the translation of policy into plans for specific actions [39].

### 2.1. Inclusion Criteria for Articles and Gray Literature

We included studies that

- were published in scientific peer-reviewed journals and gray literature for the period 2000–2014;
- were in English, French, or Italian;
- focused on descriptive/analytical epidemiology and surveillance evaluation;
- analyzed the functioning of public health surveillance systems for West Nile virus (WNV), chikungunya virus (CHKV), dengue virus (DENV), and Rift Valley fever virus (RVFV);
- reported integration between sectors.

Articles and documents were included regardless of the country/s involved in their related studies.

### 2.2. Search Strategy and Data Extraction for Articles

We defined three search axes (Table 2) and searched articles in PubMED [41] using Medical Subject Headings (MeSH) terms.

In order to identify the MeSH terms for each axis, inverse searching [37,38] was performed by analyzing the MeSH subject indexing of seven articles [22,42,43,44,45,46,47] of the desired topic, languages, and study design for WNV that also complied with the defined inclusion criteria. WNV was chosen for this analysis because it is the virus for which evidence on integrated surveillance is more likely to have been the subject of research for the longest time.

The indexing terms used for each search string were a combination of a common root with each pathogen identified in the exposure axis. The common root combined the index terms of the axes intervention and outcome (Table 3).

Following the structure provided in the PRISMA Statement [48], we organized the selection process in four phases: identification, screening, eligibility, and inclusion.

Two reviewers conducted an initial screening on articles for relevance based on title and abstracts. Following this, potentially relevant articles identified were downloaded and reviewed in full text by one of the two reviewers. When an abstract was not available, the full text of the article was downloaded to assess eligibility.

### 2.3. Search Strategy and Data Extraction for the Gray Literature

We searched for relevant gray literature published from 2000 to 2014 on the websites of ECDC and WHO (HQ, EURO, and EMRO).

All relevant documents were downloaded in full text and underwent an eligibility assessment for inclusion in the review by one reviewer.

### 2.4. Analysis

We analyzed all included articles/documents extrapolating the following information in a spreadsheet data collection grid: Exposure, Title, Authors, Journal, Year, Country (or Countries), Intervention: Surveillance (Y/N), Integrated Surveillance (Y/N), Integrated Sector(s) (human and/or animal and/or entomological and/or environmental), Level of Integration (policy and institutional level/data collection and analysis level/dissemination level), and Added Value of Integration (early warning/impact assessment/response).

## 3. Results

### 3.1. Scientific Literature

#### 3.1.1. The Selection Process

**Figure 1:**
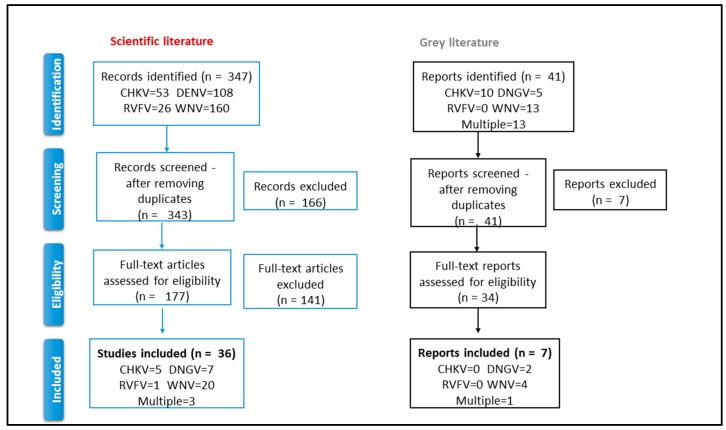
Selection process: identification, screening, eligibility, and inclusion. CHKV = chikungunya virus DENV = dengue virus WNV = West Nile virus RVFV = Rift Valley fever virus.

The extraction was conducted on the four search strings on PubMED on 29 December 2014 and led to the identification of 347 scientific articles (Table S3; PubMed DataBase). Of those, four were duplicates and were excluded. 343 articles’ titles and abstracts were assessed. For 72 of the 343 articles, the abstracts were not available, so we assessed their eligibility directly on the full texts. Based on the identified inclusion criteria, we excluded 166 articles on the basis of titles and abstracts and selected 177 (including the 72 without abstracts) for the full text assessment (Figure 1). All 177 selected full texts were available.

Thirty-five (20%) met the inclusion criteria: 20 (56%) referred to WNV, 7 (19%) to DENV, 4 (11%) to CHKV, 1 (3%) to RVFV, and 3 (8%) focused on multiple diseases (Multi). We decided to add one article (n.1 in Table S1) reporting on imported cases of CHKV because its description of the surveillance system was deemed interesting for the purpose of this research. Therefore, 36 articles were finally analyzed (Table S1).

The majority of articles (83%; 30/36) were descriptions of surveillance systems (28) or of surveillance projects (2). The rest were either studies (3) or assessments (3).

The articles reported experiences of integrated surveillance in the United States (10/36, 28%), Italy (5/36, 14%), France/Réunion Island (4/36, 11%), Canada (2/36, 6%), Cuba, Greece, Hungary, Mexico, Pacific Islands, Romania, Singapore, Spain, Trinidad and Tobago, and Turkey (1 article per country). Four articles described more than one country (4/36, 11%), while one article did not mention a specific country.

#### 3.1.2. The Integration between Sectors

All articles, except one, specified the type of sectors involved in integration (Table 4).

Eighteen (51%) reported integration across two sectors. Of these, 12 (67%) involved human and entomological sectors, and 6 involved human and animal sectors. Integration across three sectors was reported in 14/35 articles (40%), of which 12/14 (86%) reported human, animal, and entomological sector integration, and 2 reported human, entomological, and environmental sector integration. Integration across four sectors (human, animal, entomological, and environmental) was reported in 3/35 articles.

As shown in Table 5, 15 articles (56%), reported integration at all levels: policy and institutional, data collection and analysis, and dissemination.

#### 3.1.3. The Level of Integration

Out of the 35 articles mentioning the added value of integrated surveillance, early warnings, and responses was reported in 16 articles (46%, 1 CHKV, 1 DENV, 12 WNV, and 2 Multi); and the added value of early warnings, impact assessments, and responses was reported in 8 (23%, 1 CHKV, 3 DENV, 3 WNV, and 1 Multi). Five articles (14%, 2 CHKV and 3 WNV) mentioned only the added value of early warnings.

### 3.2. The Gray Literature Reports

#### 3.2.1. The Selection Process

We retrieved 41 gray literature reports: the most frequent source was ECDC (24/41, 59%) with 11/24 reports on WNV (46%) and 7/24 on CHKV (29%). While searching in WHO HQ, EURO, and EMRO websites, documents published by other regional offices were retrieved. We selected 12 reports (12/41, 29%) addressing multiple arboviral diseases (8/12, 67%), DENV (3/12, 25%), and CHKV (1/12, 8%).

Seven documents out of 41 (17%) met the inclusion criteria (Figure 1 and Table S2) reporting about the integration between surveillance systems of different sectors: WNV (4/7, 57%), DENV (2/7, 29%), and Multi (1/7).

#### 3.2.2. The Integration between Sectors

Of the six reports mentioning the type of integration, two (WNV) indicated integration across the human, animal, entomological, and environmental sectors, two (WNV) across the human, animal, and entomological sectors, and two (DENV) across the human, entomological, and environmental sectors.

Two out of six (33%) of the documents took the environmental sector into account.

#### 3.2.3. The Level of Integration

Of the five reports mentioning the level of integration, two (DENV) addressed all levels of our framework (policy and institutional, data collection and data analysis, and dissemination), one (WNV) mentioned the policy and institutional levels, one (WNV) the data collection and analysis level and the dissemination level, and one (WNV) the data collection and analysis level.

### 3.3. Lessons Learned from the Articles and Documents

In general, the articles and the documents did not report on lessons learned nor provide indications for integration strategies in the surveillance of VBD based on their experiences/studies.

Notwithstanding this, few stressed the needs of integration for a public health impact.

A joint publication of WHO and the Special Programme for Research and Training in Tropical Diseases (TDR) reported a table with examples of good and bad practices in dengue surveillance, where criteria of integration were identified among the good practices (n.3; DNG; Table S2).

Hadler et al. [49] (n.189; Multi; Table S1) reported on an assessment of WNV and other arboviral surveillance capacities that was carried out in 2012 in the United States. The mentioned selected indicators included inter-sectoral aspects. The assessment highlighted the changes that had occurred since 2004 (previous assessment) and identified the implications for public health practice.

The following articles recommended an integrated approach in the surveillance of arboviral diseases on the basis of the results of the studies/assessments described. Cito et al. [50] (n.193; Multi; Table S1), on the basis of the results of a survey conducted among six EU countries of the Mediterranean basin, recommended that “… *the surveillance of WNV and RVF must include a high level of collaboration between different professionals such as veterinarians, public health officers, entomologists and climatologists for properly dealing with vector-borne zoonotic diseases. The multi-disciplinary approach requires the establishment of integrated information systems, covering human and veterinary fields as well as providing useful data on vectors distribution and abundance*.”

Krisztalovics et al. [51] (n.243; WNV; Table S1) reported on the surveillance of WNV neuro-invasive infections in humans in Hungary: *“… the results of serological analysis used for confirmation of WNV cases are in most cases too late to apply control measures. For this purpose, it is very important to develop good collaboration with the veterinary sector to exchange information and undertake joint actions. At present the Ministry of Health and the National Centre for Epidemiology are preparing to sign an agreement with the Ministry of Agriculture, regarding collaboration with the veterinary authorities, in particular, exchange of information and vector control measures*.”

Finally, Hernández-Ávila et al., described in their article [52] (n.69; Table S1) a conceptual framework developed in Mexico for DENV surveillance, where epidemiological and entomological data are analyzed to produce risk maps that are used to target vector control activities. New epidemiological and entomological data are collected during control activities to assess their impact. This generates a knowledge database that can be used to evaluate the cost-effectiveness of control measures, accountability, and operational research.

## 4. Discussion

We conducted a scoping review with the strategic objective of reporting on criteria able to describe consistently integrated surveillance of arboviral diseases and validate a conceptual framework for studies in the context of OH.

Results on integration type confirm that integration between sectors is pathogen-driven (i.e., human, animal, and entomological for WNV, human and entomological for DENV, etc.). For this reason, assessments should focus on specific integrations that have an impact on the efficacy of surveillance systems of specific pathogens. The occasional involvement of the environmental sector reported in the surveillance of some pathogens is also a point to consider in light of environmental and climate changes with potential impact on arboviral diseases.

This finding also supports the development of conceptual frameworks that are flexible enough to account for the pathogen (arbovirus) involved and to correctly assess surveillance system integration.

Regarding the levels of integration, 22 (81%) of the 27 articles and three of the five reports mentioning levels of surveillance integration described this integration with criteria (see related column in Tables S1 and S2) comparable to those reported in the conceptual framework we proposed (Table 1). For example, almost all the papers that reported integration at the policy and institutional level mentioned also the existence of a specific national policy/plan.

Integrated systems for data collection and analysis are considered important for prevention, prediction, and control, although these systems are still rarely implemented [53,54,55]. In particular, Vrbova in 2010 [53] reports that, of 194 surveillance systems analyzed, only 36 (19%) concomitantly collected data on human and animal surveillance.

The articles and documents of this review refer to integrated data collection and analysis but often do not describe the systems in detail. In most cases, each sector seems to collect and analyze its own data, generating results that are then shared with the other sectors. This might be the reason why only 56% of the articles (14/25) reporting integration at the data collection and analysis level mention criteria of integration (e.g., the existence of a common database or data system) as proposed by our framework.

This finding is in line with the results of the survey on integrated surveillance we conducted with 19 countries of the Mediterranean and Black Sea Regions [40]. This study showed how integration mechanisms were more frequently directed to the joint dissemination of results than to the development of mechanisms/procedures for integrated data collection. This might indicate that each sector collects surveillance data separately and that results are collated in a second step for coordinated dissemination.

Although more than 80% of the articles recognized an early warning as the main added value of integrated surveillance, integration between sectors was mainly described in the context of response activities (e.g., setting up control measures). It therefore seems that prevention and mitigation of impact through early warning with an integrated inter-sectoral approach still needs attention and consolidation.

This study has of course some limits, as the use of a unique electronic database. Additionally, it should be considered that we identified criteria for integration (Tables S1 and S2) by “reading” the papers and not by doing an assessment. Therefore, if authors of selected articles/documents referred for example to “MoH Plan & Procedures for Human & Entomological Sectors” to show the integration of the system at the policy and institutional level, we assumed that the system was actually integrated at that level.

Despite these limitations, we think that the results obtained provide some indications of possible criteria to describe and evaluate integrated surveillance and help to validate the proposed framework.

## 5. Conclusions

One Health surveillance should lead to faster disease detection, more efficient disease control and tangible financial savings when formally compared against separated surveillance streams.

However, the lack of metrics and associated methods to estimate OH benefits in a systematic way is evident [56].

On the basis of the previous survey [40] and the current scoping review we have performed, we validate a conceptual framework that provides criteria against which to assess the level of integration of surveillance systems for arboviral diseases. This framework is flexible enough to cater to pathogen-driven differences in sector integration and can be used to describe and compare different integrated surveillance systems.

With the support of these criteria, additional studies should be run at the national level to compare different OH systems, to assess their cost-effectiveness, and to evaluate where the real “added value” of the sectors’ integration lies (e.g., surveillance versus response and control activities).

This could, in our view, contribute to facing present challenges in operationalizing OH and facilitating the transition from assessment to action.

In fact, it is important to identify priority areas in which to direct multi-sectoral efforts for the control of arbovirus infections. Areas of multi-sectoral collaboration can include, among others, surveillance, early warning (risk assessment, modeling, etc.) and vector control. National situation analysis can help to describe local situations and resources available, guide prioritization, and ultimately support operationalizing OH.

## Figures and Tables

**Table 1:** Proposed criteria to assess existing levels of integration.

Level of Integration	Sublevels of Integration	Criteria
Policy and institutional	Policy	1. Existence of a national policy addressing integrated surveillance for a specific pathogen
		2. Existence of a policy addressing integrated surveillance for a specific pathogen at subnational level
	Institutional	3. Existence of agreements among the institutions involved in human/animal/entomological surveillance for the specific pathogen
		4. Existence of coordination mechanisms among the institutions involved
		5. Existence of identified focal points for each human/animal/entomological surveillance for the specific pathogen
Data collection and analysis	Interoperability mechanisms at data collection	6. Existence of integrated data collection tools
		7. Existence of activation mechanisms of human surveillance based on signals from animal/entomological surveillance
		8. Other interoperability mechanisms at data collection level
	Interoperability mechanisms at data analysis	9. Presence of database exchange/merging/other mechanisms to facilitate joint analysis among sectors.
		10. Performance of joint/integrated data analysis among the different surveillance sectors
		11. Other interoperability mechanisms at data analysis level
Dissemination	-	12. Existence of joint results dissemination mechanisms (e.g., bulletins, reports, papers, media reports, websites, etc.)

**Table 2:** Review search axes.

Search Axes	Description
Intervention	Surveillance of a viral Vector Born Diseases (VBDs) that integrates human virology, animal virology and/or medical entomology components
Outcome	Models of integrated surveillance for this viral VBDs
Exposure	West Nile virus (WNV)
	chikungunya virus (CHKV)
	dengue virus (DENV)
	Rift Valley fever virus (RVFV)

**Table 3:** Review search strings.

Common Root	Exposure Disease	Strings
Intervention and Outcome	West Nile virus (WNV)	Common root and WNV
	chikungunya virus (CHKV)	Common root and CHKV
	dengue virus (DENV)	Common root and DENV
	Rift Valley fever virus (RVFV)	Common root and RVFV

**Table 4:** The number of articles reporting on the number and type of sectors integrated in surveillance, by pathogen.

Sectors	Pathogen					
	CHKV	DENV	Multi	RVFV	WNV	Total
human and entomological	5	4	2	0	1	**12**
human and animal	0	0	1	1	4	**6**
human and animal and entomological	0	0	0	0	12	**12**
human and entomological and environmental	0	2	0	0	0	**2**
human and animal and entomological and environmental	0	0	0	0	3	**3**
**Total**	**5**	**6**	**3**	**1**	**20**	**35**

**Table 5:** Number of articles reporting on the level of surveillance integration, by pathogen.

Sectors	Pathogen					
	CHKV	DENV	Multi	RVFV	WNV	Total
policy and institutional level	0	0	1	0	1	**2**
data collection and analysis level	0	3	0	0	0	**3**
data collection and analysis and dissemination levels	2	1	0	0	3	**6**
policy and institutional and data collection and analysis levels	1	0	0	0	0	**1**
policy and institutional and data collection and analysis and dissemination levels	2	1	1	0	11	**15**
**Total**	**5**	**7**	**3**	**1**	**20**	**27**

## Supplementary Materials

The following are available online at http://www.mdpi.com/1660-4601/15/3/489/s1, Table S1: Eligible articles; Table S2: Eligible documents; Table S3: PubMed database; Table S4: Scoping Review Protocol.
