# Supplementary material for: Strengthening Preparedness for Arbovirus Infections in Mediterranean and Black Sea Countries: A Conceptual Framework to Assess Integrated Surveillance in the Context of the One Health Strategy
Source: Int J Environ Res Public Health. 2018 Mar 10;15(3):489. doi: 10.3390/ijerph15030489 (PMC5877034; doi:10.3390/ijerph15030489)
Supplement: Supplementary file 1 [file ijerph-15-00489-s001.zip › ijerph-262683 supplementary/S1. eligible articles.pdf]

Table S1-Eligible articles

Ext. N	N	Exposure	Article Title	Journal: Abbreviation	Journal: Year	All Authors (Last, Initials)	Type of integration	Level of integration	criteria	Added Value	Country	Type of article
**1**	**1**	CHK	Large number of imported chikungunya cases in mainland France, 2014: a challenge for surveillance and response.	Euro Surveill	2014	Paty, MC; Six, C; Charlet, F; Heuzé, G; Cochet, A; Wiegandt, A; Chappert, JL; Dejour-Salamanca, D; Guinard, A; Soler, P; Servas, V; Vivier-Darrigol, M; Ledrans, M; Debruyne, M; Schaal, O; Jeannin, C; Helynck, B; Leparc-Goffart, I; Coignard, B	human and entomological	policy and institutional and data collection and analysis levels	MoH Plan & Procedures for Human & entomological sectors<br><br>a common system has been set up comprising entomological and epidemiological surveillance.	early warning and impact assessment and response	France	Description
**30**	**2**	CHK	Chikungunya in Europe.	Lancet	2008	Queyriaux, B; Armengaud, A; Jeannin, C; Couturier, E; Peloux-Petiot, F	human and entomological	data collection and analysis and dissemination levels	System operational	early warning	France	Description

35	**3**	CHK	Outbreak of chikungunya in the French Territories, 2006: lessons learned.	Euro Surveill	2007	Ledrans, M; Quatresous, I; Renault, P; Pierre, V	human and entomological	data collection and analysis and dissemination levels	System operational	early warning	Réunion Island	Description
36	**4**	CHK	An outbreak of chikungunya fever in the province of Ravenna, Italy.	Euro Surveill	2007	Angelini, R; Finarelli, AC; Angelini, P; Po, C; Petropulacos, K; Macini, P; Fiorentini, C; Fortuna, C; Venturi, G; Romi, R; Majori, G; Nicoletti, L; Rezza, G; Cassone, A	human and entomological	policy and institutional and data collection and analysis and dissemination levels	Regional protocol<br><br>System operational	early warning and response	Italy	Description
43	**5**	CHK	E-alert 27 January: Chikungunya outbreak in Reunion, a French overseas department.	Euro Surveill	2006	Quatresous, I; ,	human and entomological	policy and institutional and data collection and analysis and dissemination levels	Plan and Procedures available<br><br>System operational	response	Réunion Island	Description

69	**6**	DNG	Nation-wide, web-based, geographic information system for the integrated surveillance and control of dengue fever in Mexico.	PLoS One	2013	Hernández-Ávila, JE; Rodríguez, MH; Santos-Luna, R; Sánchez-Castañeda, V; Román-Pérez, S; Ríos-Salgado, VH; Salas-Sarmiento, JA	human and entomological	data collection and analysis level	System operational	early warning and impact assessment and response	Mexico	Description
90	**7**	DNG	Dengue Tools: innovative tools and strategies for the surveillance and control of dengue.	Glob Health Action	2012	Wilder-Smith, A; Renhorn, KE; Tissera, H; Abu Bakar, S; Alphey, L; Kittayapong, P; Lindsay, S; Logan, J; Hatz, C; Reiter, P; Rocklöv, J; Byass, P; Louis, VR; Tozan, Y; Massad, E; Tenorio, A; Lagneau, C; L'Ambert, G; Brooks, D; Wegerdt, J; Gubler, D		not mentioned		not mentioned	Multicountry	Project

**101**	**8**	DNG	Best practices in dengue surveillance: a report from the Asia-Pacific and Americas Dengue Prevention Boards.	PLoS Negl Trop Dis	2010	Beatty, ME; Stone, A; Fitzsimons, DW; Hanna, JN; Lam, SK; Vong, S; Guzman, MG; Mendez-Galvan, JF; Halstead, SB; Letson, GW; Kuritsky, J; Mahoney, R; Margolis, HS; ,	human and entomological	data collection and analysis level	System to be implemented	early warning and response	Multicountry	Description
134	**9**	DNG	An integrated ecosystem approach for sustainable prevention and control of dengue in Central Havana.	Int J Occup Environ Health	2006	Bonet, M; Spiegel, JM; Ibarra, AM; Kouri, G; Pintre, A; Yassi, A	human and entomological and environmental	data collection and analysis and dissemination levels	Project: pilot integrated surveillance system in place	impact assessment and response	Cuba	Project

137	**10**	DNG	Dengue prevention and 35 years of vector control in Singapore.	Emerg Infect Dis	2006	Ooi, EE; Goh, KT; Gubler, DJ	human and entomological and environmental	policy and institutional and data collection and analysis and dissemination levels	National strategy and integrated system recommended	early warning and impact assessment and response	Singapore	Description
140	**11**	DNG	Impact of vector control on a dengue fever outbreak in Trinidad, West Indies, in 1998.	Trop Med Int Health	2005	Chadee, DD; Williams, FL; Kitron, UD	human and entomological	data collection and analysis level	Not mentioned	impact assessment	Trinidad	Description
157	**12**	DNG	Dengue surveillance in the Pacific Islands.	Pac Health Dialog	2000	Condon, R; Taleo, G; Stewart, T; Sweeney, T; Kiedrzynski, T	human and entomological	not mentioned	------------	early warning and impact assessment and response	Pacific Islands	Description

180	**13**	RVF	Outbreaks of Rift Valley fever in Kenya, Somalia and United Republic of Tanzania, December 2006-April 2007.	Wkly Epidemiol Rec	2007		human and animal	not mentioned	----------------	impact assessment and response	Multicountry	Description
191	**14**	WNV	West Nile virus and non-West Nile virus mortality and coinfection of American crows (Corvus brachyrhynchos) in California.	Avian Dis	2014	Wheeler, SS; Woods, LW; Boyce, WM; Eckstrand, CD; Langevin, SA; Reisen, WK; Townsend, AK	human and animal	data collection and analysis and dissemination levels	Study: not mentioned	early warning and impact assessment and response	United States	Study

189	**15**	Multi	National capacity for surveillance, prevention, and control of West Nile virus and other arbovirus infections--United States, 2004 and 2012.	MMWR Morb Mortal Wkly Rep	2014	Hadler, JL; Patel, D; Bradley, K; Hughes, JM; Blackmore, C; Etkind, P; Kan, L; Getchell, J; Blumenstock, J; Engel, J; ,	human and entomological	policy and institutional level	National Plan and Cooperative Agreements	early warning and impact assessment and response	United States	Assessment
5	**16**	Multi	Human and entomological surveillance of West Nile fever, dengue and chikungunya in Veneto Region, Italy, 2010-2012.	BMC Infect Dis	2014	Gobbi, F; Capelli, G; Angheben, A; Giobbia, M; Conforto, M; Franzetti, M; Cattelan, AM; Raise, E; Rovere, P; Mulatti, P; Montarsi, F; Drago, A; Barzon, L; Napoletano, G; Zanella, F; Pozza, F; Russo, F; Rosi, P; Palù, G; Bisoffi, Z; ,	human and entomological	policy and institutional and data collection and analysis and dissemination levels	Regional Plan System in place	early warning and response	Italy	Description

192	**17**	WNV	Integrated human surveillance systems of West Nile virus infections in Italy: the 2012 experience.	Int J Environ Res Public Health	2013	Napoli, C; Bella, A; Declich, S; Grazzini, G; Lombardini, L; Nanni Costa, A; Nicoletti, L; Pompa, MG; Pupella, S; Russo, F; Rizzo, C	human and animal and entomological and environmental	policy and institutional and data collection and analysis and dissemination levels	National Plan	early warning and impact assessment and response	Italy	Description

193	**18**	Multi	Analysis of surveillance systems in place in European Mediterranean countries for West Nile virus (WNV) and Rift Valley fever (RVF).	Transbound Emerg Dis	2013	Cito, F; Narcisi, V; Danzetta, ML; Iannetti, S; Sabatino, DD; Bruno, R; Carvelli, A; Atzeni, M; Sauro, F; Calistri, P	human and animal	not mentioned	---------------	early warning and response	Multicountry	Assessment
199	**19**	WNV	The 2012 West Nile encephalitis epidemic in Dallas, Texas.	JAMA	2013	Chung, WM; Buseman, CM; Joyner, SN; Hughes, SM; Fomby, TB; Luby, JP; Haley, RW	human and entomological	not mentioned	-------------------	early warning and response	United States	Study

211	**20**	WNV	Emergence of West Nile virus infections in humans in Turkey, 2010 to 2011.	Euro Surveill	2012	Kalaycioglu, H; Korukluoglu, G; Ozkul, A; Oncul, O; Tosun, S; Karabay, O; Gozalan, A; Uyar, Y; Caglayık, DY; Atasoylu, G; Altas, AB; Yolbakan, S; Ozden, TN; Bayrakdar, F; Sezak, N; Pelıtlı, TS; Kurtcebe, ZO; Aydın, E; Ertek, M	human and animal and entomological	policy and institutional and data collection and analysis and dissemination levels	National Plan	impact assessment and response	Turkey	Description
212	**21**	WNV	Epidemiological surveillance of West Nile neuroinvasive diseases in Italy, 2008 to 2011.	Euro Surveill	2012	Rizzo, C; Salcuni, P; Nicoletti, L; Ciufolini, MG; Russo, F; Masala, R; Frongia, O; Finarelli, AC; Gramegna, M; Gallo, L; Pompa, MG; Rezza, G; Salmaso, S; Declich, S	human and animal and entomological	policy and institutional and data collection and analysis and dissemination levels	National Plan	early warning and response	Italy	Description

216	**22**	WNV	West Nile fever outbreak in horses and humans, Spain, 2010.	Emerg Infect Dis	2011	García-Bocanegra, I; Jaén-Téllez, JA; Napp, S; Arenas-Montes, A; Fernández-Morente, M; Fernández-Molera, V; Arenas, A	human and animal and entomological	policy and institutional and data collection and analysis and dissemination levels	National Plan	early warning	Spain	Description
233	**23**	WNV	Ongoing outbreak of West Nile virus infections in humans in Greece, July-August 2010.	Euro Surveill	2010	Papa, A; Danis, K; Baka, A; Bakas, A; Dougas, G; Lytras, T; Theocharopoulos, G; Chrysagis, D; Vassiliadou, E; Kamaria, F; Liona, A; Mellou, K; Saroglou, G; Panagiotopoulos, T	human and animal and entomological	policy and institutional and data collection and analysis and dissemination levels	National Plan Website dissemination	early warning and response	Greece	Description

236	**24**	WNV	West Nile virus circulation in Emilia-Romagna, Italy: the integrated surveillance system 2009.	Euro Surveill	2010	Angelini, P; Tamba, M; Finarelli, AC; Bellini, R; Albieri, A; Bonilauri, P; Cavrini, F; Dottori, M; Gaibani, P; Martini, E; Mattivi, A; Pierro, AM; Rugna, G; Sambri, V; Squintani, G; Macini, P	human and animal and entomological	policy and institutional and data collection and analysis and dissemination levels	Regional Plan Integrated dissemination	early warning and response	Italy	Description
243	**25**	WNV	West Nile virus infections in Hungary, August-September 2008.	Euro Surveill	2008	Krisztalovics, K; Ferenczi, E; Molnar, Z; Csohan, A; Ban, E; Zoldi, V; Kaszas, K	human and animal	policy and institutional and data collection and analysis and dissemination levels	National Plan	early warning and response	Hungary	Description

263	**26**	WNV	A GIS-driven integrated real-time surveillance pilot system for national West Nile virus dead bird surveillance in Canada.	Int J Health Geogr	2006	Shuai, J; Buck, P; Sockett, P; Aramini, J; Pollari, F	human and animal	not mentioned	----------------	early warning	Canada	Description
280	**27**	WNV	Assessment of surveillance of human West Nile virus infection in Quebec, 2003.	Can Commun Dis Rep	2004	Gaulin, C; Couillard, M; Pilon, PA; Tremblay, M; Lambert, L; Fradet, MD; Deschênes, L; Fortin, A; Poulin, C	human and animal and entomological	data collection and analysis and dissemination levels	computerized system in place	early warning and response	Canada	Assessment
281	**28**	WNV	Department of Defense West Nile virus surveillance in 2002.	Mil Med	2004	Witt, CJ; Brundage, M; Cannon, C; Cox, K; Clements, TE; Cooper, ED; Elbert, Y; Ludwig, GV; Mangiafico, JA; Malakooti, M; Miller, MK; Osborn, SD; Pagac, B; Ross, LP; Shelton, LJ; Spring, A; Kelley, P	human and animal	data collection and analysis and dissemination levels	Not mentioned	early warning and response	United States	Description

296	**29**	WNV	Surveillance results from the first West Nile virus transmission season in Florida, 2001.	Am J Trop Med Hyg	2003	Blackmore, CG; Stark, LM; Jeter, WC; Oliveri, RL; Brooks, RG; Conti, LA; Wiersma, ST	human and animal and entomological	policy and institutional and data collection and analysis and dissemination levels	Not mentioned	early warning and impact assessment and response	United States	Study
321	**30**	WNV	West Nile virus: a case study in how NY State Health Information infrastructure facilitates preparation and response to disease outbreaks.	J Public Health Manag Pract	2001	Gotham, IJ; Eidson, M; White, DJ; Wallace, BJ; Chang, HG; Johnson, GS; Napoli, JP; Sottolano, DL; Birkhead, GS; Morse, DL; Smith, PF	human and animal and entomological	policy and institutional and data collection and analysis and dissemination levels	National Plan Web-based exchange of electronic information<br><br>Dissemination formats	impact assessment and response	United States	Description

327	**31**	WNV	West Nile virus surveillance in Connecticut in 2000: an intense epizootic without high risk for severe human disease.	Emerg Infect Dis	2001	Hadler, J; Nelson, R; McCarthy, T; Andreadis, T; Lis, MJ; French, R; Beckwith, W; Mayo, D; Archambault, G; Cartter, M	human and animal and entomological	policy and institutional and data collection and analysis and dissemination levels	Different databased used in the analysis	early warning and response	United States	Description
331	**32**	WNV	West Nile virus 2000-2001.	N J Med	2001	Ziskin, LZ	human and animal and entomological and environmental	not mentioned	-------------	early warning	United States	Description
332	**33**	WNV	West Nile virus: New Jersey's 2000 experience and surveillance plans for 2001.	N J Med	2001	Farello, CA; Sorhage, FE; Bresnitz, EA; Grant, C	human and animal and entomological and environmental	policy and institutional and data collection and analysis and dissemination levels	Integrated working group; Regular meetings; Integrated database; Publication on CDC’s website	early warning and response	United States	Description

337	**34**	WNV	The West Nile virus: its recent emergence in North America.	Microbes Infect	2001	Garmendia, AE; Van Kruiningen, HJ; French, RA	human and animal and entomological	not mentioned	-----------------	early warning and response	United States	Description
338	**35**	WNV	West nile virus surveillance in Romania: 1997-2000.	Viral Immunol	2001	Ceianu, CS; Ungureanu, A; Nicolescu, G; Cernescu, C; Nitescu, L; Tardei, G; Petrescu, A; Pitigoi, D; Martin, D; Ciulacu-Purcarea, V; Vladimirescu, A; Savage, HM	human and animal and entomological	policy and institutional level	Not mentioned	early warning and response	Romania	Description
341	**36**	WNV	Outbreak not contained. West Nile virus triggers a reevaluation of public health surveillance.	Sci Am	2000	Holloway, M	human and animal and entomological	not mentioned	---------------	early warning and response	NA-overview	Description
