# Supplementary material for: Strengthening Preparedness for Arbovirus Infections in Mediterranean and Black Sea Countries: A Conceptual Framework to Assess Integrated Surveillance in the Context of the One Health Strategy
Source: Int J Environ Res Public Health. 2018 Mar 10;15(3):489. doi: 10.3390/ijerph15030489 (PMC5877034; doi:10.3390/ijerph15030489)
Supplement: Supplementary file 1 [file ijerph-15-00489-s001.zip › ijerph-262683 supplementary/S2.eligible documents.pdf]

Table S2- Eligible documents

n	Source	Exposure	title	Author/s	Year	surveillance	integration	type of integration	levels	criteria
3	WHO HQ	DNG	Dengue: guidelines for diagnosis, treatment, prevention and control. New edition.	A joint publication of WHO and the Special Programme for Research and Training in Tropical Diseases (TDR)	2009	yes	yes	human and entomological and environmental Dengue surveillance: examples of good and bad practice	policy and institutional and data collection and analysis and dissemination levels	information exchange on data and analysis surveillance and monitoring of environmental between agencies involved in disease and social risks surveillance, vector surveillance and the monitoring of environmental and social risks
5	WHO/CDC	Multi	Technical Guidelines for Integrated Disease Surveillance and Response in the African Region		2010	yes	yes	human and animal and environmental	not mentioned	
7	WHO HQ	DNG	Global strategy for dengue prevention and control 2012-2020.		2012	yes	yes	human and entomological and environmental	policy and institutional and data collection and analysis and dissemination levels	integrated information system multisectoral dengue action committee an emergency action plan
24	ECDC	WNV	West Nile virus risk assessment tool		2013	yes	yes	human and animal and entomological and environmental	policy and institutional level	not mentioned

			Stockholm: ECDC; 2013.							
25	ECDC	WNV	West Nile virus infection in Greece in 2012		2012	yes	yes	human and animal and entomological and environmental	policy and institutional level<br><br>data collection and analysis level	The Ministry of Health is currently developing a National Action Plan for vector-borne diseases, including specific sections on malaria, West Nile virus and other vector-borne diseases. The objective is that this National Plan will be endorsed by all involved ministries and provide the necessary legal framework for assigning responsibilities to all stakeholders.<br><br>centralised web platform (MALWEST project)
30	ECDC	WNV	Expert consultation on West Nile virus infection		2011	yes	yes	human and animal and entomological	data collection and analysis and dissemination levels	not mentioned
34	ECDC	WNV	Expert consultation on West Nile virus infection		2009	yes	yes	human and animal and entomological	not mentioned	----------------
