# Supplementary material for: Strengthening Preparedness for Arbovirus Infections in Mediterranean and Black Sea Countries: A Conceptual Framework to Assess Integrated Surveillance in the Context of the One Health Strategy
Source: Int J Environ Res Public Health. 2018 Mar 10;15(3):489. doi: 10.3390/ijerph15030489 (PMC5877034; doi:10.3390/ijerph15030489)
Supplement: Supplementary file 1 [file ijerph-15-00489-s001.zip › ijerph-262683 supplementary/S4 Scoping Review Protocol.pdf]

Review Protocol

Contents

Review Protocol 1
Contents 2
1. Background 3
2. Study question 3
3. Objectives 3
3.1 General Aim 3
3.2 Specific objectives 3
4. Methods 3
4.1 Study selection, search restrictions, inclusion and exclusion criteria 3
4.3 Search strategy for the identification of articles ad reports 6
4.4 Document selection and data extraction procedure 9
4.4 Data synthesis procedure 11
5. References 11

1. Background

Coordination of epidemiological surveillance in the three specific areas of human and animal virology and medical entomology is considered a crucial step to facilitate data exchange and data management and ultimately improve prevention and control of arboviral zoonotic diseases as addressed by the One Health approach.

In the framework of the EC-DEVCO funded project MediLabSecure, Istituto Superiore di Sanità (ISS-Italy) is tasked of assessing the level of integration between animal virology, human virology, medical entomology and human surveillance in Countries of the Mediterranean and Black Sea Regions.

In order to do this, there is the need to clearly define what is integrated surveillance/one health surveillance in the field of arboviral diseases. Criteria are needed to describe and define this integration in a consistent way.

2. Study question

Which criteria can be used to define integrated surveillance (human, animal, entomological) in the field of autochthonous vector borne viral diseases in countries of the Mediterranean Black Sea and EU regions?

3. Objectives

3.1 General Aim

To identify criteria able to consistently describe integrated surveillance of autochthonous arboviral diseases to be used for studies in the context of One Health .

3.2 Specific objectives

- To analyse studies describing existing surveillance systems of autochthonous vector borne viral diseases, including systems successfully integrating human, animal virology and/or medical entomology surveillance.
- To identify those elements that should be present in order to defined a surveillance system as "integrated" (hereby criteria).

4. Methods

The methodology of this review has been defined taking into account the methodology for the conduction of systematic and scoping literature reviews proposed by Khan et al [1], [2]the first VBORNET project strategic paper [3],and search strategies as presented by Relevo et al.[4] and by DeLuca et al.[5].

Given the specificity of integrated surveillance activities to the different pathogens, there is a need to define the search strategy of this review in a way to analyse surveillance integration by disease. There is therefore the need to identify which VBD should be considered in this review on the basis of those most relevant to the geographical area of interest of the MediLabSecure Project.

The following viral vector borne diseases have caused autochthonous cases (endemic/sporadic) in the EU, Mediterranean and Black Sea regions: West Nile Virus (WNV) [6], Chikungunya [7,8], Dengue [9,10], Rift Valley Fever (RVF) [11], Crimean Congo Haemorrhagic Fever (CCHF) [12], Tick Borne Encephalitis (TBE) [13, 14].

As the current priority of the MediLabSecure Project has been narrowed down to mosquito transmitted VBD, this review will consider the following diseases: WNV, Chikungunya, Dengue and RVF.

4.1 Study selection, search restrictions, inclusion and exclusion criteria

4.1.1 Selection: Studies and reports pertinent and relevant to describing the **functioning of surveillance systems** for WNV, Chikungunya, Dengue, RVF.

4.1.2 Search Restrictions: Publication date from 2000 to present, publication language in English, French, Italian.

4.1.3 Inclusion: Descriptive/analytical epidemiology and surveillance evaluation studies/reports analyzing the functioning of public health surveillance systems for WNV, Chickungunya, Dengue, RVF and reporting integration between sectors.

4.1.4 Exclusion: Documents for which abstracts/full texts are not retrievable from open source and journal subscriptions available through the Italian Institute of Public Health.

4.2 Selection of documents and flow of information through the different phases of the systematic literature review

Following the structure provided in the PRISMA Statement [15], the document selection process to be conducted in this literature review is structured in four phases: identification, screening, eligibility and inclusion (Figure 1).

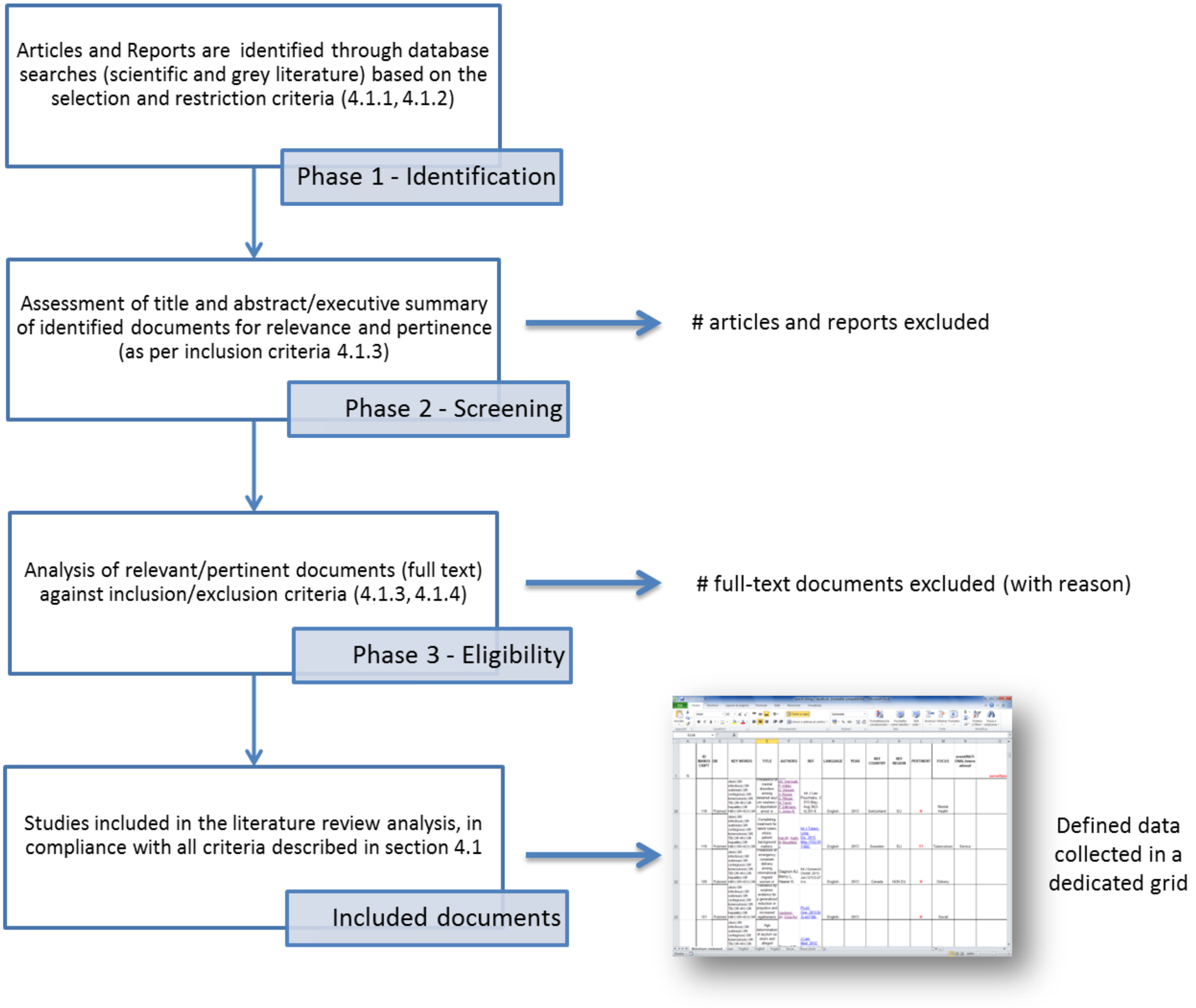

Figure 1 – Selection of documents and flow of information through the different phases of the systematic literature review (adapted from the PRISMA Statement [15])

4.3 Search strategy for the identification of articles ad reports

This review will combine a scientific literature review (section 4.3.2) with a grey literature review (section 4.3.3), both conducted through an automated process using online databases/websites.

Three search axes have been defined: **Intervention, Outcome and Exposure** (Table 1). A common search root will be defined for the first two axes: Intervention and Outcome. This common root will be then combined in turn to each disease identified under the third axis: Exposure.

Table 1 – Review search axes

Search Axes	Description
Intervention	Surveillance of a viral VBD that integrates human virology, animal virology and/or medical entomology components
Outcome	models of integrated surveillance for this viral VBD
Exposure	West Nile Virus
	Chikungunya
	Dengue
	Rift Valley Fever

4.3.1 Indexing terms

The first step in developing the automated search was identifying for each axes Medical Subject Headings (MeSH) terms.

In order to do this, inverse searching was performed by analysing the MeSH subject indexing of seven articles of the desired topic, languages and study design for exposure WNV that also complied with the defined inclusion criteria. WNV was chosen for this analysis because it is the disease for which evidence on integrated surveillance is likely to have been object of research for the longest time.

Four papers described integrated human/animal/entomological surveillance of WNV in Italy [16, 17, 18, 19], two WNV veterinary surveillance, in Greece [20] and Tunisia [21], and one human WNV surveillance in the United States of America [22]. One of the Italian papers also described surveillance of Dengue and Chikungunya.

Major and non-major topic MeSH headings associated with these articles were identified for each search axis. Following this, each potential MeSH heading was evaluated in the National Library of Medicine's MeSH database examining entry term, scope note, subheadings and related terms. Index words for each exposure was derived by searching each disease defined in the exposure axis in the MeSH database and applying the index pattern observed for WNV.

Using the embedded "PubMed Search Builder" function a final pool of search terms was defined.

Finally, additional search terms taking into account title headings, translations in the three inclusion languages and currently used acronyms were identified for use in the grey literature search (Table 3).

Table 2 – MeSH and indexing terms by axes

Search Axis	MeSH terms (headings and subheadings)	Additional search terms (Grey Literature)
Intervention	((((((("Public Health Surveillance/methods"[Mesh]) OR ("Population Surveillance/methods"[Mesh] OR "Population Surveillance/organization and administration"[Mesh] OR "Population Surveillance/standards"[Mesh] OR "Population Surveillance/statistics and numerical data"[Mesh] OR "Population Surveillance/utilization"[Mesh] OR "Population Surveillance/veterinary"[Mesh])) OR "Incidence"[Mesh]) OR ("Data Collection/epidemiology"[Mesh] OR "Data Collection/veterinary"[Mesh])) OR ("Public Health/epidemiology"[Mesh] OR "Public Health/statistics and numerical data"[Mesh] OR "Public Health/veterinary"[Mesh])) OR "Sentinel Surveillance"[Mesh]) OR ("Disease Outbreaks/prevention and control"[Mesh] OR "Disease Outbreaks/statistics and numerical data"[Mesh])) OR "Epidemiological Monitoring"[Mesh]	"surveillance" OR "notification OR "monitoring" OR "sorveglianza" OR "notifica"
Outcome	"Animals"[Mesh]) OR "Humans"[Mesh])	"human" OR "animal" OR "entomological" OR "humain" OR "animale" OR "umana" OR "entomologica" OR "entomologique" OR "integration" OR "integration" OR integrazione" OR "integrated" OR "intégrée" OR "integrata"
Exposure	("West Nile Fever/epidemiology"[Majr] OR "West Nile Fever/prevention and control"[Majr] OR "West Nile Fever/statistics and numerical data"[Majr] OR "West Nile Fever/veterinary"[Majr])	"WNV" OR "Febbre del Nilo" OR "fièvre du Nil occidental" OR "FNO"
	("Alphavirus Infections/epidemiology"[Majr] OR "Alphavirus Infections/prevention and control"[Majr] OR "Alphavirus Infections/statistics and numerical data"[Majr] OR "Alphavirus Infections/veterinary"[Majr])	"Chickungunya" OR "CHK"
	("Dengue/epidemiology"[Majr] OR "Dengue/prevention and control"[Majr] OR "Dengue/statistics and numerical data"[Majr] OR "Dengue/veterinary"[Majr])	"Dengue" OR "Dengue Haemorrhagic Fever" OR "DHF" OR "Dengue Fever" OR "DF" OR "dengue hémorragique" OR "fièvre de dengue" OR "FD" OR "DH"
	("Rift Valley Fever/epidemiology"[Majr] OR "Rift Valley Fever/prevention and control"[Majr] OR "Rift Valley Fever/statistics and numerical data"[Majr] OR "Rift Valley Fever/veterinary"[Majr])	"Rift Valley Fever" OR "RVF" OR "Fièvre de la Vallée du Rift" OR "FVR" OR "Febbre del Nilo Occidentale"
	("Hemorrhagic Fever, Crimean/epidemiology"[Majr] OR "Hemorrhagic Fever, Crimean/prevention and control"[Majr] OR "Hemorrhagic Fever, Crimean/statistics and numerical data"[Majr] OR "Hemorrhagic Fever, Crimean/veterinary"[Majr])	"CCHF" OR "Fièvre hémorragique de Crimée Congo" OR "FHCC" OR "febbre Congo-Crimea"
	("Encephalitis, Tick-Borne/epidemiology"[Majr] OR "Encephalitis, Tick-Borne/prevention and control"[Majr] OR "Encephalitis, Tick-Borne/statistics and numerical data"[Majr] OR "Encephalitis, Tick-Borne/veterinary"[Majr])	"TBE" OR "TBEV" OR "encéphalite à tique" OR "méningoencéphalite à tique" OR "MET" OR "meningoencefalite da zecca" OR "encefalite da zecca"

4.3.2 Scientific literature search strategy

Search for relevant scientific literature will be performed on the PubMED [23]. The database will be searched using MeSH terms.

As described in the search strategy, the indexing terms used for each search stream will be a combination of a common root with each exposure disease. The common root combines the index terms of the axes intervention and outcome. Language and publication date restrictions are included as specified in the inclusion criteria listed in section 4.1 of this protocol (Box 1).

Box -1 Example of the combined common (in italic), exposure specific (in bold) inclusion criteria related (green) PubMED search for WNV

((((((("Public Health Surveillance/methods"[Majr]) OR ("Population Surveillance"[Majr] OR "Population Surveillance/organization and administration"[Majr] OR "Population Surveillance/standards"[Majr] OR "Population Surveillance/statistics and numerical data"[Majr] OR "Population Surveillance/utilization"[Majr] OR "Population Surveillance/veterinary"[Majr])) OR ("Public Health/epidemiology"[Majr] OR "Public Health/veterinary"[Majr])) OR "Sentinel Surveillance"[Majr]) OR ("Disease Outbreaks/prevention and control"[Majr]) OR "Epidemiological Monitoring"[Majr]) AND ("Animals"[Mesh] OR "Humans"[Mesh])) AND ("West Nile Fever/epidemiology"[Majr] OR "West Nile Fever/prevention and control"[Majr] OR "West Nile Fever/statistics and numerical data"[Majr] OR "West Nile Fever/veterinary"[Majr])** AND ("01/01/2000"[Date - Create] : "3000"[Date - Create]))) AND (English[Language] OR Italian[Language] OR French[Language])

Box -2 Example of the combined common (in italic), exposure specific (in bold) inclusion criteria related (green) PubMED search for Chickungunya

((((((("Public Health Surveillance/methods"[Majr]) OR ("Population Surveillance"[Majr] OR "Population Surveillance/organization and administration"[Majr] OR "Population Surveillance/standards"[Majr] OR "Population Surveillance/statistics and numerical data"[Majr] OR "Population Surveillance/utilization"[Majr] OR "Population Surveillance/veterinary"[Majr])) OR ("Public Health/epidemiology"[Majr] OR "Public Health/veterinary"[Majr])) OR "Sentinel Surveillance"[Majr]) OR ("Disease Outbreaks/prevention and control"[Majr]) OR "Epidemiological Monitoring"[Majr]) AND ("Animals"[Mesh] OR "Humans"[Mesh])) AND ("Alphavirus Infections/epidemiology"[Majr] OR "Alphavirus Infections/prevention and control"[Majr] OR "Alphavirus Infections/statistics and numerical data"[Majr] OR "Alphavirus Infections/veterinary"[Majr])** AND ("01/01/2000"[Date - Create] : "3000"[Date - Create]))) AND (English[Language] OR Italian[Language] OR French[Language])

Box -3 Example of the combined common (in italic), exposure specific (in bold) inclusion criteria related (green) PubMED search for Dengue

((((((("Public Health Surveillance/methods"[Majr]) OR ("Population Surveillance"[Majr] OR "Population Surveillance/organization and administration"[Majr] OR "Population Surveillance/standards"[Majr] OR "Population Surveillance/statistics and numerical data"[Majr] OR "Population Surveillance/utilization"[Majr] OR "Population Surveillance/veterinary"[Majr])) OR ("Public Health/epidemiology"[Majr] OR "Public Health/veterinary"[Majr])) OR "Sentinel Surveillance"[Majr]) OR ("Disease Outbreaks/prevention and control"[Majr]) OR "Epidemiological Monitoring"[Majr]) AND ("Animals"[Mesh] OR "Humans"[Mesh])) AND ("Dengue/epidemiology"[Majr] OR "Dengue/prevention and control"[Majr] OR "Dengue/statistics and numerical data"[Majr] OR "Dengue/veterinary"[Majr])** AND ("01/01/2000"[Date - Create] : "3000"[Date - Create]))) AND (English[Language] OR Italian[Language] OR French[Language])

Box --4? Example of the combined common (in italic), exposure specific (in bold) inclusion criteria related (green) PubMED search for RVF

((((((("Public Health Surveillance/methods"[Majr]) OR ("Population Surveillance"[Majr] OR "Population Surveillance/organization and administration"[Majr] OR "Population Surveillance/standards"[Majr] OR "Population Surveillance/statistics and numerical data"[Majr] OR "Population Surveillance/utilization"[Majr] OR "Population Surveillance/veterinary"[Majr])) OR ("Public Health/epidemiology"[Majr] OR "Public Health/veterinary"[Majr])) OR "Sentinel Surveillance"[Majr]) OR ("Disease Outbreaks/prevention and control"[Majr]) OR "Epidemiological Monitoring"[Majr]) AND ("Animals"[Mesh] OR "Humans"[Mesh])) **AND ("Rift Valley Fever/epidemiology"[Majr] OR "Rift Valley Fever/prevention and control"[Majr] OR "Rift Valley Fever/statistics and numerical data"[Majr] OR "Rift Valley Fever/veterinary"[Majr]) AND ("01/01/2000"[Date - Create] : "3000"[Date - Create]))) AND (English[Language] OR Italian[Language] OR French[Language])**

4.3.3 Grey literature search strategy

Search for relevant grey literature will be performed on the ECDC and WHO (HQ, EURO, EMRO) websites. The search will be subject based by Exposure axis. Syntax will be adapted to the websites searched.

4.4 Document selection and data extraction procedure

As shown in Figure 1, following the identification of articles through the described automated literature searches, the first step of selection will be the **screening** of abstracts/executive summaries for relevance and pertinence to the selection criteria.

All relevant/pertinent documents will be downloaded in full text and will undergo an **eligibility assessment** for inclusion in this review by two reviewers.

All included articles will match the criteria listed in section 4.1 of this protocol and will be analysed extrapolating the following information included in a data collection grid:

- Title
- Authors
- Journal
- Year
- Country
- Type of Study/Report
- Exposure (WNV/Chikungunya/Dengue/RVF/)
- What is integrated (human and/or animal and/or entomological and/or environmental)
- How it is integrated (policy and institutional level/ data collection and analysis level/ dissemination level)
- Added value of integration (early warning/impact assessment/response)
- Criteria utilised to report the level of integration

For each document analysed in full text, the criteria proposed in Table 4 to describe existing levels of integration will be assessed.

Table 3 – Proposed criteria to describe existing levels of integration between human/animal/entomological surveillance for a specific exposure

Level of integration	Sublevels of integration	Criteria
Policy and institutional level	Policy level	1. Existence of a National policy addressing integrated surveillance for this specific exposure<br>2. Existence of a policy addressing integrated surveillance for this specific exposure at subnational level
	Institutional level	3. Existence of agreements among the institutions involved in human/animal/entomological surveillance for the specific exposure,<br>4. Existence of a coordination mechanisms among the institutions involved,<br>5. Existence of identified focal points for each of human/animal/entomological surveillance for the specific exposure
Data collection and analysis level	Interoperability mechanisms at data collection level	6. Existence of integrated data collection tools<br>7. Existence of activation mechanisms of human surveillance based on signals from animal/entomological surveillance<br>8. Other interoperability mechanisms at data collection level
	Interoperability mechanisms at data analysis level	9. Presence of DB exchange/merging/other mechanisms to facilitate joint analysis among sectors.<br>10. Performance of joint/integrated data analysis among the different surveillance sectors<br>11. Other interoperability mechanisms at data analysis level
Dissemination level	-	12. Existence of joint result dissemination mechanisms (e.g. bulletins, reports, papers, media reports, websites …)

4.5 Data synthesis procedure

The findings will be presented in tables structured as shown by exposure.

Table 4 – Dummy Table of surveillance integration type, exposure x

No Integration (N. %)	Integration (N. %)				
	Animal/human (N., %, countries)	Entomological/human (N., %, countries)	Animal/entomological (N., %, countries)	Animal/human/entomological (N., %, countries)	Other (N., %, countries)
xx	xx	xx	xx	xx	xx

Table 5 – Dummy Table of surveillance integration level, exposure x

No Integration (N. %)	Integration (N. %)		
	Policy and institutional level (N., %, countries)	Data collection and analysis level (N., %, countries)	Dissemination level (N., %, countries)
xx	xx	xx	xx

The declared integration in relation to the criteria will be also described.

5. References

[1] Khan KS, Kunz R, Kleijnen J, Antes G. *Five steps to conducting a systematic review.* J R Soc Med. Mar 2003; 96(3): 118–121. Available at http://www.ncbi.nlm.nih.gov/pmc/articles/PMC539417/#__ffn_sectitle

[2] Arksey H. and O'Malley L. Scoping studies: towards a methodological framework International Journal of Social Research Methodology: Theory & Practice, Vol. 8, No. 1, 02.2005, p. 19-32. Available from http://eprints.whiterose.ac.uk/1618/1/Scopingstudies.pdf (latest access 18/07/2016)

[3] Braks M., Van der Giessen J., Kretzschmar M. et al. *Towards an integrated approach in surveillance of vector-borne diseases in Europe"* Pararsites & Vectors 2011, 4:192

[4] Relevo R. *Effective Search Strategies for Systematic Reviews of Medical Tests* in Methods Guide for Medical Test Reviews [Internet]. Chang SM, Matchar DB, Smetana GW, et al., editors. Available at http://www.ncbi.nlm.nih.gov/books/NBK98242/
Rockville (MD): Agency for Healthcare Research and Quality (US); 2012 Jun.

[5] DeLuca JB, Mullins MM, Lyles CM, Crepaz N, Kay L, Thadiparthi S. *Developing a comprehensive strategy for evidence based systematic reviews.* Evidence based library and information practice 2008, 3:1

[6] ECDC. *Distribution of West Nile Fever cases by affected areas, European Region/Mediterranean Basin. Transmission Season 2013*. Available at http://www.ecdc.europa.eu/en/healthtopics/west_nile_fever/West-Nile-fever-maps/Pages/historical-data.aspx

[7] Grandadam M, Caro V, Plumet S. et al. *Chikungunya Virus, Southeastern France*. Emerg Infect Dis. May 2011; 17(5): 910–913. doi: 10.3201/eid1705.101873 available at http://www.ncbi.nlm.nih.gov/pmc/articles/PMC3321794/#!po=8.33333

[8] Seyler T, Rizzo C, Finarelli AC, Po C, Alessio P, Sambri V, Ciofi Degli Atti ML, Salmaso S. *Autochthonous chikungunya virus transmission may have occurred in Bologna, Italy, during the summer 2007 outbreak*. Euro Surveill. 2008;13(3):pii=8015. Available at http://www.eurosurveillance.org/ViewArticle.aspx?ArticleId=8015.

[9] Marchand E, Prat C, Jeannin C, et al. *Autochthonous case of dengue in France, October 2013.* Euro Surveill. 2013;18(50):pii=20661. Available online: http://www.eurosurveillance.org/ViewArticle.aspx?ArticleId=20661

[10] Schaffner F, Fontenille D, Mathis A. Autochthonous dengue emphasises the threat of arbovirosis in Europe. Lancet Infect Dis. 2014 Sep 10. pii: S1473-3099(14)70925-9.

[11] Balenghien T, Cardinale E, Chevalier V et al. *Towards a better understanding of Rift Valley fever epidemiology in the south-west of the Indian Ocean.* Vet Res. 2013; 44(1): 78. Available online at http://www.ncbi.nlm.nih.gov/pmc/articles/PMC3848763/#__ffn_sectitle

[12] Maltezou HC, Papa A. *Crimean-Congo hemorrhagic fever: epidemiological trends and controversies in treatment*. BMC Medicine 2011, 9:131 BMC Medicine 2011, 9:131. Available online at http://download.springer.com/static/pdf/183/art%253A10.1186%252F1741-7015-9-131.pdf?auth66=1416222920_c7926b778c22114fdd4235d59a432a6f&ext=.pdf

[13] ECDC Technical Report Epidemiological situation of tick-borne encephalitis in the European Union and European Free Trade Association countries, 2012. Available at http://www.ecdc.europa.eu/en/publications/Publications/TBE-in-EU-EFTA.pdf

[14] Fischer M, Yendell S.J., Rollin P.E. *Tickborne Encephalitis* in CDC Yellow Book 2014 available at http://wwwnc.cdc.gov/travel/yellowbook/2014/chapter-3-infectious-diseases-related-to-travel/tickborne-encephalitis

[15] Moher D., Liberati A., Tetzlaff J., Altman D.G., The PRISMA Group. Preferred Reporting Items for Systematic Reviews and Meta-Analyses: the PRISMA statement" PLoS Med. 2009 Jul; 6(7) e1000097

[16] Napoli C, Bella A, Declich S et. Al. *Integrated human surveillance systems of West Nile virus infections in Italy: the 2012 experience*. Int J Environ Res Public Health. 2013 Dec 13;10(12):7180-92.

[17] Gobbi F, Capelli G, Angheben A et al. *Human and entomological surveillance of West Nile fever, dengue and chikungunya in Veneto Region, Italy, 2010-2012.* BMC Infect Dis. 2014 Feb 5;14:60.
[18] Rizzo C, Salcuni P, Nicoletti L et al. *Epidemiological surveillance of West Nile neuroinvasive diseases in Italy, 2008 to 2011.* Euro Surveill. 2012 May 17;17(20).
[19] Angelini P, Tamba M, Finarelli AC et al. *West Nile virus circulation in Emilia-Romagna, Italy: the integrated surveillance system 2009.* Euro Surveill. 2010 Apr 22;15(16).
[20] Chaintoutis SC, Dovas CI, Papanastassopoulou M et al. *Evaluation of a West Nile virus surveillance and early warning system in Greece, based on domestic pigeons.* Comp Immunol Microbiol Infect Dis. 2014 Mar;37(2):131-41.
[21] Ben Hassine T1, Hammami S, Elghoul H, Ghram A. *Detection of circulation of West Nile virus in equine in the north-west of Tunisia.* Bull Soc Pathol Exot. 2011 Oct;104(4):266-71.
[22] Lindsey NP1, Staples JE, Lehman JA et al. *Surveillance for human West Nile virus disease - United States, 1999-2008.* MMWR Surveill Summ. 2010 Apr 2;59(2):1-17.
[23] PubMED website http://www.ncbi.nlm.nih.gov/pubmed
